# Minimally Invasive Management of Acute Biliary Tract Disease during Pregnancy

**DOI:** 10.1155/2009/829020

**Published:** 2009-07-12

**Authors:** Luis Tomás Chiappetta Porras, Eduardo Daniel Nápoli, Carlos Manuel Canullán, Bernabé Matías Quesada, Hernán Eduardo Roff, Juan Alvarez Rodríguez, Alejandro Salvador Oría

**Affiliations:** ^1^Department of Surgery, “Cosme Argerich” Hospital, Almirante Brown 240, Buenos Aires 1155, Argentina; ^2^University of Buenos Aires, Paraguay 2155, Buenos Aires 1121, Argentina

## Abstract

*Background*. Acute biliary diseases during pregnancy have been classically managed conservatively. Advances in minimally invasive surgery and the high recurrence rate of symptoms observed changed this management. *Methods*. This is a prospective observational study. Initial management was medical. Unresponsive patients were treated with minimally invasive techniques including gallbladder percutaneous aspiration or cholecystostomy, endoscopic retrograde cholangiography, and laparoscopic cholecystectomy, depending on the pregnancy trimester and underlying diagnosis. *Results*. 122 patients were admitted. 69 (56.5%) were unresponsive to medical treatment. Recurrent gallbladder colic was the most frequent indication for minimally invasive intervention, followed by acute cholecystitis, choledocholithiasis, and acute biliary pancreatitis. 8 patients were treated during the first trimester, 54 during the second, and 7 during the last trimester. There was no fetal morbidity or mortality. Maternal morbidity was minor with no mortality. *Conclusion*. Acute biliary tract diseases during pregnancy may be safely treated with minimally invasive procedures according to the underlying diagnosis and to the trimester of pregnancy.

## 1. Introduction

Symptomatic biliary tract disease during pregnancy has been traditionally managed conservatively, leaving surgery for treatment failures. A recent report suggests that fetal death rate is higher after conservative treatment than after laparoscopic cholecystectomy for symptomatic benign biliary disease [1]. Recurrence rates after conservative treatment seem to be trimester depending and range between 40–92% [1, 2].

Surgical advances made over the last two decades shifted the management towards a multidisciplinary and minimally invasive approach. Interventional radiology, endoscopic retrograde cholangiography (ERCP), and laparoscopic surgery are commonly employed, but their timing and indications need to be properly defined.

The objective of this study is to analyze the results obtained with a minimally invasive protocol to treat patients with acute biliary tract disease during pregnancy.

## 2. Material and Methods

From January 1998 through January 2008 all patients with acute benign biliary tract disease during pregnancy were enrolled in a prospective treatment protocol. Inclusion diagnoses were gallbladder colic, cholecystitis, choledocholithiasis, and acute biliary pancreatitis.

Exclusion criteria were as follows.

- Severe cardiac disorders such as congestive heart failure, diagnosis of unstable angina, or acute myocardial infarction.
- Severe impairment of renal function requiring dialysis.
- Coagulopathy or medical affection requiring anticoagulation.
- Mental incapacity, unwillingness, or language barriers precluding adequate understanding or cooperation or compromises the ability of the subject to give written informed consent.

All diagnoses were made with a combination of medical history and physical examination, laboratory tests (such as amylase in the case of acute pancreatitis) and ultrasonography. Diagnosis of gallbladder colic was made with the combination of biliary pain in the presence of gallbladder lithiasis and in the absence of ultrasonographic signs of gallbladder inflammation. Diagnosis of acute cholecystitis was made when biliary pain was associated with the presence of both gallbladder lithiasis and inflammation (wall edema, gallbladder distension, etc.). Acute biliary pancreatitis was defined with the presence of biliary pain, elevated serum amylase, presence of gallbladder lithiasis, and ultrasonographic signs of pancreatic inflammation. Choledocholithiasis was defined with the presence of biliary symptoms, jaundice, abnormal liver function tests, presence of gallbladder lithiasis and a common bile duct diameter greater than 7 mm.

All patients were initially managed conservatively. Conservative management varied according with the admission diagnosis. Patients unresponsive or relapsing after medical treatment were considered conservative treatment failures and were treated using minimally invasive procedures.

Pregnant patients presenting with recurrent gallbladder colic during the first or third trimester were managed with percutaneous gallbladder aspiration. Patients presenting with acute cholecystitis during the same period of pregnancy were treated with percutaneous cholecystostomy. Those patients presenting with biliary obstruction during the first or third trimester of pregnancy were initially managed with ERCP. Definitive treatment in all cases was done at the postpartum period with laparoscopic cholecystectomy and single stage management of choledocholithiasis when required, following a protocol previously described [3].

During the second trimester of pregnancy (13° to 33° week) patients were managed with laparoscopic surgery. SAGES guidelines [4] were strictly followed and included pneumatic compression of the lower extremities, peroperative fetal monitoring, intraoperative maternal oxicapnography, uterine protection with a lead shield, open-access neumoperitoneum and working pressures between 8 and 12 mm Hg. Uterine inhibition with ritodrine was administered to all patients for the first 36 hours.

All patients received adequate information regarding signs and symptoms of possible complications or relapse of their pathology and were strongly counseled to visit the hospital for consultation. Additional information provided by the nutritionist was given, in order to avoid certain foods that could be responsible for new symptomatic episodes.

The local ethics committee approved the protocol and all patients signed an informed consent prior to their inclusion in the present study.

## 3. Results

A total of 122 patients were enrolled in the treatment protocol. Admission diagnoses were recurrent gallbladder colic in 55 cases, acute cholecystitis in 41, bile duct obstruction in 18, and acute biliary pancreatitis in 8.75% of patients were admitted initially at our hospital and 25% were referred from other institutions. All patients were referred after initial consultation to the emergency department. Mean age was 24 years (range 15–39 years).

Conservative treatment failure occurred in 69 patients (56.5%). 8 patients were treated during the first trimester of pregnancy of whom 4 with acute cholecystitis were treated with percutaneous cholecystostomy, 3 admitted because of recurrent gallbladder colics were treated with percutaneous gallbladder aspiration with symptomatic relief in all of them and one patient with acute biliary pancreatitis was treated with ERCP removing 2 bile duct stones. 54 patients were admitted during the second trimester of pregnancy and were treated with laparoscopic surgery. Mean gestational age was 24.6 weeks. Admission diagnoses were recurrent gallbladder colic in 20 cases, acute cholecystitis in 17, bile duct obstruction in 12 and acute biliary pancreatitis in 5. All cases underwent laparoscopic cholecystectomy with intraoperative cholangiography; 21 (39%) had common bile duct stones. 16 were successfully treated with a transcystic approach and the remaining 5 required laparoscopic choledocotomy because of multiple bile duct stones. After complete bile duct clearance all these cases were finished with primary duct closure. Mean hospitalization time was 1.8 days (range 1–3 days) for laparoscopic cholecystectomy and 3 days (range 2–4 days) for common bile duct exploration. 7 patients were treated in the last trimester. 3 required ERCP with stent placement because of multiple common bile duct stones. 3 required percutaneous gallbladder aspiration because of recurrent gallbladder colics and 1 because of acute cholecystitis. 96.5% of patients were followed for a medium time of 60 months (range 2–120 months). There were no fetal morbidity or mortality. Maternal morbidity included 2 minor wound infections after laparoscopic surgery managed with oral antibiotics in an outpatient basis. There was no maternal mortality.

## 4. Discussion

Acute benign biliary diseases and acute appendicitis are the most common surgical indications during pregnancy [5]. Swisher et al. [2] demonstrated that the risk of symptoms recurrence after successful medical treatment for acute biliary diseases was 92% in the first trimester, 64% in the second trimester, and 44% in the last one. Recurrent episodes of biliary pancreatitis were observed in 50% of patients after conservative treatment [6]. In Ghummann's series [7] the most common cause of biliary surgery during pregnancy were repeated biliary colic in 70% of cases, followed by acute cholecystitis in 20%, choledocholithiasis in 7% and acute biliary pancreatitis in the remaining 3% of cases. In the present series the most common cause of surgery was also recurrent biliary colic with 37.5%, followed by acute cholecystitis with 32% of cases.

At the beginning of laparoscopic era, cholecystectomy during pregnancy was considered as a relative contraindication, mainly because of the lack of knowledge of the effects of CO_2_ to the fetus. Fear of surgical treatment was based on the potential risk of abortion or malformations if done during the first trimester or preterm labor when done in the last one. It is therefore recommended treatment during these periods to be done with percutaneous or endoscopic techniques. Today, there is unanimous agreement concerning the security of laparoscopic cholecystectomy during the second trimester of pregnancy and some extent the indication to the first and third trimester too [7–11]. However, our protocol includes laparoscopic surgery only during second trimester, using minimally invasive techniques such as ERCP, percutaneous cholecystostomy or percutaneous gallbladder aspiration for patients in the first or second trimester. The absence of morbidity and mortality with these techniques in our series is similar as reported by others [12, 13].

Potential risks of laparoscopic surgery during pregnancy are uterine lesion during trocar placement, induction of preterm labor and fetal acidosis, among many others. Peroperative fetal monitoring as indicated in SAGES guidelines is extremely important in preventing the last two potential complications mentioned. However, a recent study by Lu et al. [14] reports a higher incidence of spontaneous or induced preterm labor for patients with conservative versus surgical treatment, with a clear relation with symptoms recurrence. Even though the risks to the fetus during the second trimester for radiation exposure are low, it is recommended to protect the uterus with a lead shield. Similarly, working pressures of CO_2_ are recommended to be below 12 mm Hg in order to prevent fetal acidosis [4].

Known benefits of laparoscopic surgery are the lower necessity of narcotic use in the postoperative period, a lower risk of wound-related complications, and a faster return to ambulation and oral intake, among many others. Curet et al. [5] support this in pregnant patients, demonstrating a significant reduction in hospitalization time for laparoscopic cases in comparison between open and laparoscopic cholecystectomy, although this was a retrospective one, with the inheriting biases of this kind of studies.

In conclusion, our results suggest that laparoscopic cholecystectomy with or without bile duct exploration can be carried out safely in pregnant patients with acute biliary tract diseases unresponsive to medical treatment between weeks 13–33. Patients during the first or final trimester should be treated with less invasive procedures such as endoscopic or percutaneous techniques. These results might allow us to change the original protocol and now patients treated with endoscopic or percutaneous techniques during the first trimester could be definitively managed with laparoscopic cholecistectomy in the second trimester instead of waiting until the postpartum period.
